# Comorbidity Between Crowned Dens Syndrome and Primary Hyperparathyroidism

**DOI:** 10.7759/cureus.40831

**Published:** 2023-06-22

**Authors:** Soki Tamiya, Ryo Ichibayashi, Sadamu Takahashi, Hayato Hanada, Shiho Nakano

**Affiliations:** 1 Division of Emergency Medicine, Department of Internal Medicine, Toho University Medical Center, Sakura Hospital, Chiba, JPN

**Keywords:** cervical spine computed tomography, pathological fracture, hyperparathyroidism, hypercalcemia, crowned dens syndrome

## Abstract

Primary hyperparathyroidism (PHPT) is characterized by various symptoms, including malaise, psychiatric symptoms, and hypertension. When hypercalcemia is accompanied by PHPT, it may cause pathologic fractures or lethargy. Additionally, PHPT can be complicated by crowned dens syndrome (CDS).

We present a case of a 72-year-old female. She had begun experiencing low back pain during movement five days before. The symptoms progressed and the patient was unable to move. Based on the imaging, blood tests, and clinical findings, the patient was diagnosed with PHPT complicated with CDS.

Therefore, it is important to perform a careful physical examination of the neck and cervical spine computed tomography in patients with PHPT. Moreover, measuring calcium levels in patients with CDS may lead to early detection of PHPT.

## Introduction

Crowned dens syndrome (CDS) is an inflammatory condition induced by calcium pyrophosphate dihydrate crystal deposition in the atlantoaxial joint. It commonly occurs in older women and is characterized by a rapid onset of symptoms, severely restricted range of motion, neck pain, and increased inflammatory reaction. Cervical spine computed tomography (CT) in these patients reveals calcification around the odontoid process. Additionally, calcium pyrophosphate dihydrate crystal deposition disease has been associated with primary hyperparathyroidism (PHPT) [1]. However, no detailed reports have been published to date regarding the clinical course of patients diagnosed with PHPT complicating CDS or those diagnosed with CDS complicating PHPT. The reports diagnosing CDS do not measure parathyroid hormone or calcium levels. Testing parathyroid hormone and calcium levels for CDS may help diagnose and treat missed PHPT at an early stage. Here, we report the clinical characteristics of a patient with CDS complicating PHPT, who was diagnosed based on a detailed examination for consciousness disturbance.

## Case presentation

The patient was a 72-year-old without a significant past or family history. She had no pressure ulcers prior to onset and was grade 1 on the modified Rankin Scale. She had back pain that did not interfere with her life. She did not take drugs regularly or have a history of trauma. She had begun experiencing low back pain during movement five days prior. The symptoms progressed and the patient was unable to move as well as eat or drink water. A family member visited her home and found her with urinary and fecal incontinence. She was thus transported to our hospital by ambulance. Upon arrival, her consciousness was E3V4M6 on the Glasgow coma scale. Moreover, her body temperature was 37.2℃; respiration rate, 18/min; pulse rate, 116/min; blood pressure, 195/125 mmHg; and SpO2 (room air), 96%. No abnormal findings were observed in the chest and abdomen. Neck pain, associated restriction of range of motion, and thoracolumbar spinal tapping pain were observed. She had pressure ulcers on the left cheek, left anterior chest, right elbow, and pubic area. Electrocardiography revealed sinus rhythm with no other abnormal findings. Blood tests showed an increased inflammatory reaction with C-reactive protein (CRP) of 15.45 mg/dL. Further, she presented electrolyte imbalance with Sodium 156 mEq/L and Calcium 12.7 mg/dL. Blood urea nitrogen and creatine levels were 68.8 and 0.88 mg/dL, respectively (Table 1).

**Table 1:** Laboratory results upon admission CRP: C-reactive protein, TP: total protein, Alb: albumin, AST: aspartate aminotransferase, ALT: alanine aminotransferase, LDH: lactate dehydrogenase, ALP: alkaline phosphatase, γ-GTP: γ-glutamyl transpeptidase, D-Bil: direct bilirubin, BUN: blood urea nitrogen,  eGFR: estimated glomerular filtration, IP: inorganic phosphorus, PT-INR: prothrombin time-international normalized ratio, APTT: activated partial thromboplastin time, 1α25(OH)2VitD: 1α25-dihydroxy-vitaminD, PTHintact: parathyroid hormone intact, PTHrPintact: parathyroid hormone-related peptide intact.

Test	Result	Unit	Reference range
CRP	15.45	mg/dL	<0.3
TP	8.2	g/dL	6.7-8.3
ALB	3.8	g/dL	3.8-5.2
AST	39	IU/L	10-40
ALT	48	IU/L	5-45
LDH	254	U/L	124-222
ALP	86	U/L	38-113
γ-GTP	40	IU/L	<30
D-Bil	1.5	mg/dL	0.2-1.2
BUN	68.8	mg/dL	8.0-20.0
Creatinine	0.88	mg/dL	0.47-0.79
eGFR	48	mL/min/1.73m^2^	
Uric acid	13.4	mg/dL	2.5-7.0
Sodium	156	mEq/L	137-147
Potassium	3.5	mEq/L	3.5-5.0
Chlorine	115	mEq/L	98-108
Calcium	12.7	mg/dL	8.4-10.4
IP	2.9	mg/dL	2.5-4.5
Magnesium	2.8	mg/dL	1.9-2.5
Glucose	119	mg/dL	70-109
PT-INR	0.99		0.85-1.15
APTT	25.2	Sec	25.1-36.5
1α25(OH)_2_VitD	51	pg/mL	20-60
PTHintact	165	pg/mL	10-65
PTHrPintact	<1.1	pmol/L	<1.1

**Figure 1:**
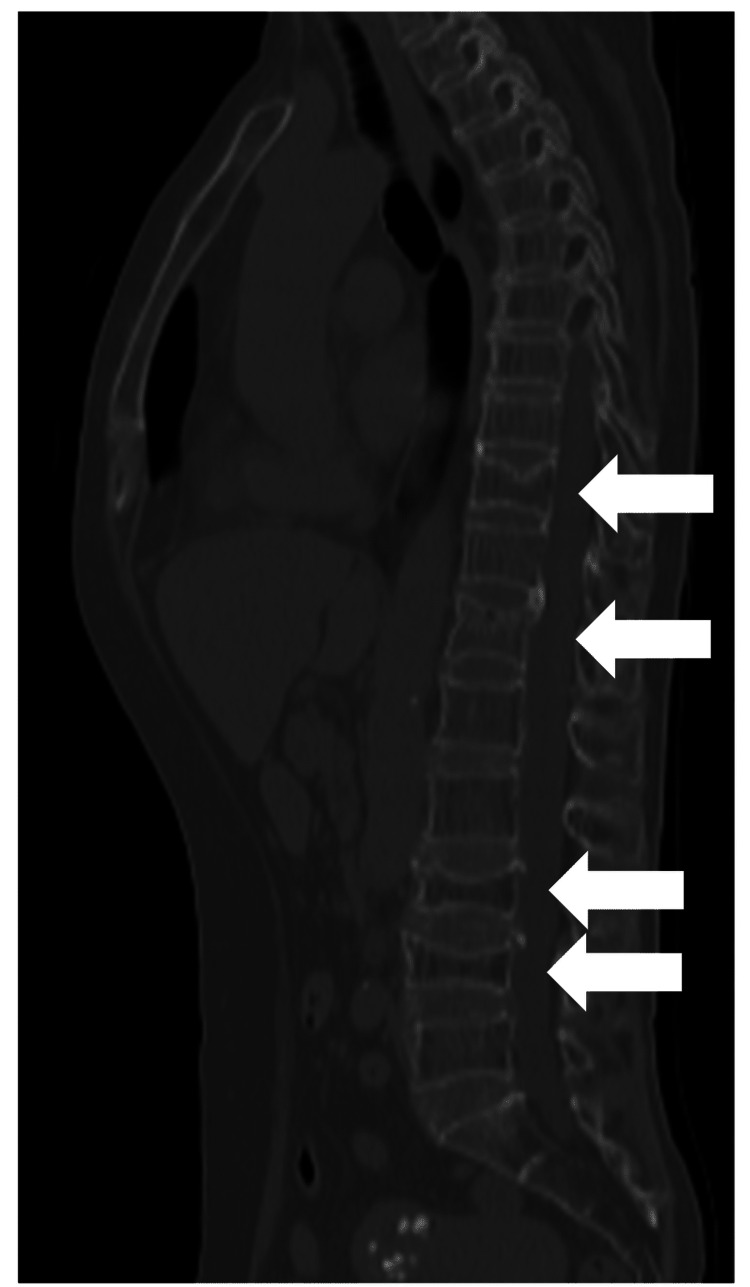
Spinal CT image A compression fracture can be observed in Th10, Th12, L3, and L4 (white arrows).

To evaluate the complaints of lethargy, neck pain, and low back pain, a whole-body CT, including the neck, chest, and pelvis, was performed (Figure 1).

**Figure 2:**
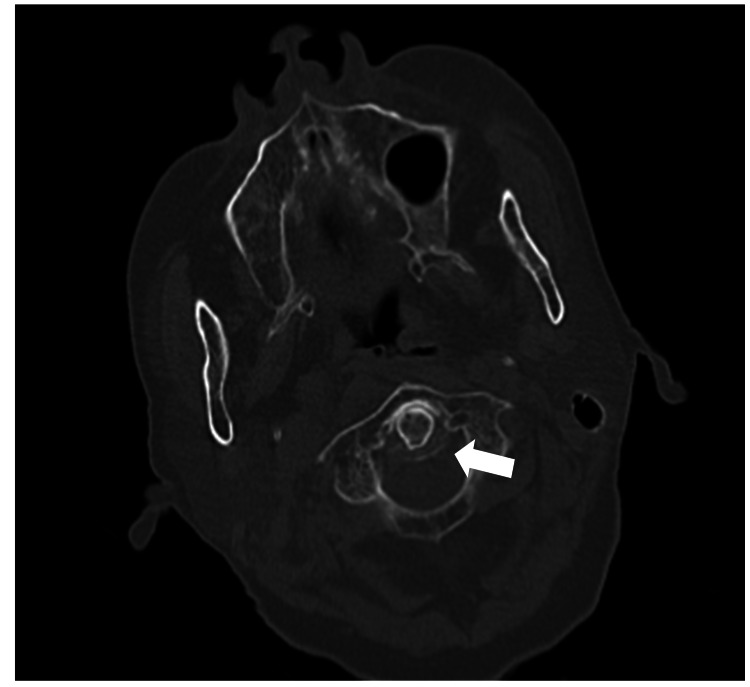
Cervical spine CT image Calcification around the second cervical odontoid process (white arrow).

No intracranial hemorrhage was detected. Cervical spine CT revealed calcification around the odontoid process of the 2nd cervical vertebra (Figure 2).

Based on the imaging and clinical findings, she was diagnosed with CDS. Additionally, multiple thoracolumbar compression fractures were observed. This was the cause of her back pain. There were no abnormal findings in the lung field, mediastinum, or abdominal cavity. Sputum, blood, and urine samples were collected and cultured to determine an increased inflammatory reaction and fever status. Accordingly, the patient was started on ceftriaxone 2 g/day. All cultures were negative, so antibiotics were discontinued. The high inflammatory response was considered to be the effect of CDS. And hypernatremia, hypercalcemia, and dehydration may have caused consciousness disturbance. Therefore, fluid infusion was used to correct the electrolyte imbalance and dehydration. The patient's consciousness became clearer as the hypercalcemia improved. Thoracic spine compression fractures were conservatively treated using a corset and acetaminophen administration. On hospital day 5, her CRP level decreased to 6.76 mg/dL. Since urine and blood cultures were negative, antibiotic therapy was discontinued, and an increased inflammatory reaction was thus judged to be due to CDS in this patient. On hospital day 8, her intact parathyroid hormone (PTH) was 165 pg/mL; 1α.25(OH)2 vitamin D, 51 pg/mL; and intact PTH-related protein, < 1.1 pmol/L ‘Table 1’. Moreover, the test for the Bence-Jones protein was negative. The urinary Ca/Cr level was 0.0386 mg/gCr, indicating a decrease in renal excretion of Ca. Based on the above blood and urinary tests, we considered it unlikely that a disease other than PHPT was the cause of hypercalcemia. The bone mineral density of the lumbar spine and femoral neck are shown in Table 2.

**Table 2:** The bone mineral density of the lumbar spine and femoral neck

Site	Bone mineral density	Young adult mean (YAM)	T score
Lumbar spine 1-3	0.513 g/cm^2^	51 %	-4.6
Femoral neck	0.454 g/cm^2^	58 %	-3.1

The Young adult mean (YAM) was less than 70% and T score less than -2.5, which was bone density sufficient for the diagnosis of osteoporosis. A thyroid ultrasound showed a mass in the right lobe suspicious of parathyroid glands. Parathyroid MIBI (99mTc-methoxy-isobutyl-isonitrile) scan showed no abnormal accumulation of parathyroid adenoma or suspected cancer. Based on the aforementioned findings, the patient has PHPT complicated with osteoporosis and several fractures. She also has CDS which may be secondary to PHPT. Rehabilitation was started on hospital day 9. Following blood tests and imaging studies, evocalcet 1 mg was started on hospital day 23. Thereafter, she was transferred to a rehabilitation hospital, with no evidence of recurrent hypercalcemia, on hospital day 41.

## Discussion

This case was initially diagnosed with multiple lumbar compression fractures and CDS. Furthermore, as consciousness disturbance was confirmed, the calcium level was measured. As a result, she was diagnosed with PHPT. PHPT is responsible for the development of pseudogout in 4.9% of all cases. Pseudogout and CDS are caused by the calcium pyrophosphate dihydrate crystal formation in joints [2]. Although the factors that regulate calcium pyrophosphate dihydrate crystal formation remain unclear, high levels of cartilage extracellular pyrophosphate are required for calcium pyrophosphate dihydrate crystal formation. Moreover, high local levels of calcium could be involved in calcium pyrophosphate dihydrate crystal formation, which is associated with PHPT [1]. For this reason, PHPT is commonly cited as a cause of CDS; however, it remains unclear what percentage of all cases of CDS are secondary to PHPT [1]. This is because the reports diagnosing CDS do not measure calcium levels.

CDS is characterized by an acute onset of neck and shoulder stiffness as well as an increased inflammatory reaction induced by calcium pyrophosphate dihydrate crystal deposition on the odontoid process of the cervical spine [3]. CDS is mainly diagnosed based on clinical symptoms and imaging findings [4,5]. Since cervical spine radiography alone might miss the presence of axial linear calcification, the diagnosis of CDS may be delayed if cervical spine CT is not performed. Most cases of CDS are idiopathic. For this reason, clinicians rarely investigate the cause of CDS. And, it is difficult to recognize PHPT unless blood calcium levels are measured.

In our patient, PHPT and CDS were suspected because the blood test results indicated hypercalcemia and CT revealed multiple compression fractures of the spine and calcification around the second cervical odontoid process. Therefore, carefully evaluating cervical spine imaging findings and calcium levels may improve the diagnostic rates of both CDS and PHPT.

Calcium level measurement is important in this case; approximately 22%-80% of patients with PHPT present with asymptomatic hypercalcemia [6]. Therefore, the incidental detection of high serum calcium levels may raise suspicion of PHPT [7]. And, in patients with hypercalcemia, a diagnosis of PHPT is established by a marked increase in PTH levels. However, evaluating serum calcium levels requires meticulous attention because some patients with PHPT may have normal PTH or normal corrected calcium levels despite elevated PTH levels [8], which may delay the diagnosis. Therefore, CDS patients should measure both calcium and PTH levels.

On the other hand, considering the possibility that CSD is likely to occur secondary to conditions, such as PHPT, it is important to assess blood calcium levels and perform a whole-body CT (the neck to the pelvis), in addition to checking for calcification around the odontoid process on cervical spine CT and an increased inflammatory reaction.

## Conclusions

If there is no hypercalcemia in CDS, the effect of parathyroid hormone is not considered. It should be noted, however, that some PHPTs may have normal calcium levels. On the other hand, imaging studies are not performed on patients with PHPT who do not have definite neck pain. The possibility of comorbid CDS and PHPT should always be considered. Therefore, when a PHPT patient complains of neck pain, it is important to perform a careful physical examination of the neck and cervical spine computed tomography. Moreover, measuring calcium levels in patients with CDS may lead to early detection of PHPT.

## References

[1] Kleiber Balderrama C, Rosenthal AK, Lans D, Singh JA, Bartels CM (2017). Calcium pyrophosphate deposition disease and associated medical comorbidities: a national cross-sectional study of US veterans. Arthritis Care Res (Hoboken).

[2] Rosenthal AK (2021). Calcium pyrophosphate deposition and crowned dens syndrome. Cleve Clin J Med.

[3] Godfrin-Valnet M, Godfrin G, Godard J, Prati C, Toussirot E, Michel F, Wendling D (2013). Eighteen cases of crowned dens syndrome: presentation and diagnosis. Neurochirurgie.

[4] Chang EY, Lim WY, Wolfson T, Gamst AC, Chung CB, Bae WC, Resnick DL (2013). Frequency of atlantoaxial calcium pyrophosphate dihydrate deposition at CT. Radiology.

[5] Haikal A, Everist BM, Jetanalin P, Maz M (2020). Cervical CT-dependent diagnosis of crowned Dens syndrome in calcium pyrophosphate dihydrate crystal deposition disease. Am J Med.

[6] Fraser WD (2009). Hyperparathyroidism. Lancet.

[7] Silverberg SJ, Bilezikian JP (1996). Evaluation and management of primary hyperparathyroidism. J Clin Endocrinol Metab.

[8] Khan AA, Hanley DA, Rizzoli R (2017). Primary hyperparathyroidism: review and recommendations on evaluation, diagnosis, and management. A Canadian and international consensus. Osteoporos Int.

